# Disease causing mutations in inverted formin 2 regulate its binding to G-actin, F-actin capping protein (CapZ α-1) and profilin 2

**DOI:** 10.1042/BSR20150252

**Published:** 2016-02-29

**Authors:** Ruth Rollason, Matthew Wherlock, Jenny A. Heath, Kate J. Heesom, Moin A. Saleem, Gavin I. Welsh

**Affiliations:** * Academic Renal Unit, School of Clinical Sciences, University of Bristol, Dorothy Hodgkin Building, Whitson Street, Bristol BS1 3NY, U.K.

**Keywords:** actin cytoskeleton, FSGS, podocytes, proteomics

## Abstract

Focal segmental glomerulosclerosis (FSGS) is a devastating form of nephrotic syndrome which ultimately leads to end stage renal failure (ESRF). Mutations in inverted formin 2 (INF2), a member of the formin family of actin-regulating proteins, have recently been associated with a familial cause of nephrotic syndrome characterized by FSGS. INF2 is a unique formin that can both polymerize and depolymerize actin filaments. How mutations in INF2 lead to disease is unknown. In the present study, we show that three mutations associated with FSGS, E184K, S186P and R218Q, reduce INF2 auto-inhibition and increase association with monomeric actin. Furthermore using a combination of GFP–INF2 expression in human podocytes and GFP-Trap purification coupled with MS we demonstrate that INF2 interacts with profilin 2 and the F-actin capping protein, CapZ α-1. These interactions are increased by the presence of the disease causing mutations. Since both these proteins are involved in the dynamic turnover and restructuring of the actin cytoskeleton these changes strengthen the evidence that aberrant regulation of actin dynamics underlies the pathogenesis of disease.

## Abstract (precis)

Mutations in inverted formin 2 (INF2) cause focal segmental glomerulosclerosis (FSGS) a major cause of end-stage kidney disease. In the present study, we show that disease associated mutations reduce INF2 auto-inhibition and cause increased binding to monomeric G-actin, profilin 2 and the F-actin capping protein, CapZ α-1.

## INTRODUCTION

The glomerulus is the filtration unit of the kidney and is composed of a bundle of capillaries which are highly permeable to water, and yet are able to selectively allow passage of solutes while retaining larger macromolecules. This selectivity is achieved through the action of the glomerular filtration barrier which consists of the glomerular endothelial cells, glomerular basement membrane and podocytes. Podocytes are terminally differentiated epithelial cells that are critical in preventing protein passage across the filtration barrier. Podocytes have branching and interdigitating processes, and filtration takes place through slits between these processes. The maintenance of its specific cell morphology is essential for the proper functioning of podocytes and this is largely dependent on a highly dynamic underlying network of protein scaffolding. The shape of this cytoskeleton is dictated by a number of regulatory factors, and disruption of function can lead to a failure to form an appropriate cell shape, which in turn can lead to a disease state [1]. Focal and segmental glomerulosclerosis (FSGS) is a major cause of end-stage kidney disease. Recent advances in molecular genetics show that defects in the podocyte play a major role in the pathogenesis of FSGS [2–4] and that mutations in inverted formin 2 (INF2), a member of the formin family of actin-regulating proteins, cause autosomal dominant FSGS [5–7].

INF2 is a member of the diaphanous subfamily of formins and has a similar domain architecture to other related subfamily members. The N-terminal half comprises a predominantly regulatory function and encompasses an overlapping Rho binding domain and diaphanous inhibitory domain (DID). The C-terminal half includes formin homology domains 1 and 2 (FH1 and FH2) and a diaphanous auto-regulatory domain (DAD). The DAD domain also acts as a monomeric G-actin binding Wiskott–Aldrich syndrome homology region 2 (WH2 domain).

INF2 is unique among the formin family members in that it possesses a dual catalytic activity with regard to actin dynamics. As well as nucleating actin filaments and promoting their elongation, INF2 can also accelerate F-actin depolymerization and filament severing [8]. This severing and depolymerization activity appears to be dependent on the activity of both the FH2 and WH2 domains acting in concert [9]. Regulation of other members of the diaphanous-related subfamily of formins is achieved through interaction of the DID and DAD domains, constraining the formin in a closed conformation which inhibits its actin nucleating and polymerizing activities. This auto-inhibition is at least partially relieved through binding of active, GTP-bound Rho GTPases to the N-terminal DID domain. Unusually, in the case of INF2, DID–DAD interaction does not inhibit actin polymerization but does inhibit actin depolymerization and severing [9].

In T-lymphocytes INF2 regulates MAL-mediated transport of the src-family kinase lymphocyte-specific protein tyrosine kinase Lck to the plasma membrane [10]. INF2 functions in the regulation of basolateral-to-apical transcytosis and lumen formation, perinuclear actin assembly and in an actin-dependent step in mitochondrial fission [11–13]. In cultured podocytes INF2 has been reported to regulate cellular actin dynamics by antagonizing Rho/diaphanous-related formin signalling and disease causing mutations have been shown to alter this signalling in the glomerulus [14,15]. INF2 has been shown to bind to and be regulated by the Rho-GTPase CDC42 in a GTP-loaded-dependent manner via its DID domain although there is a question as to whether this is a direct interaction [11,16,17]. The mutant forms of INF2 show increased binding to cdc42.

We studied three disease causing mutations in INF2 (E184K, S186P and R218Q) that all lie within the DID. We show that these mutations reduce INF2 auto-inhibition by weakening the interaction between the DID and DAD domains leading to an increased association with monomeric actin. Further we demonstrate an interaction of INF2 with profilin 2 and the F-actin capping protein, CapZ α-1 both of which are increased by the presence of the disease causing mutations.

## MATERIALS AND METHODS

### Co-immuoprecipitation and pulldowns

pET32a–INF2–DAD was made by PCR cloning using 2850–3750 of the coding region of INF2. The construct was also FLAG tagged at the N-terminus. The pGEX4T3–INF2–DID–WT,–E184K,–R218Q and–S186P constructs were made by PCR using 4–1029 of the INF2 coding region and the constructs were HA tagged at the C-terminus. All constructs were transformed into Rosetta cells (Novagen) for protein expression. Constructs were grown in 100 ml (His-tagged proteins) or 50 ml (HA-tagged proteins) of LB and protein expression induced with 100 mM IPTG, pelleted and resuspended in 5 ml lysis buffer [PBS, 1% Triton TX-100, 1 mM PMSF, protease inhibitor cocktail (Sigma), 10 mM imidazole]. Cells were lysed by sonication, pelleted and the His-tagged protein supernatants were incubated with HisPur Cobalt resin (Pierce) with 2% BSA for 30 min at 4°C. Beads were washed 5× with lysis buffer (plus an extra 100 mM sodium chloride), resuspended and snap frozen in liquid N_2_, each aliquot having 10–20 μg of protein linked to the beads. Aliquots of agarose bead linked INF2–DAD and control were incubated with HA-tagged DID domain lysates for 1 h at 4°C, beads were then washed 5× with wash buffer (PBS + extra 100 mM sodium chloride, 1 mM PMSF), boiled for 10 min with sample buffer, separated by PAGE, transferred to PVDF and probed with an anti-HA antibody.

For co-immunoprecipitation (co-IP) of endogenous protein 5 mg of polyclonal INF2 antibody or Rabbit Ig (Bethyl Labs or Millipore) was incubated with protein A/G agarose beads overnight, the beads were washed and incubated with lysates from differentiated podocytes [10 mM Tris pH 7.5, 150 mM sodium chloride, 0.5 mM EDTA, 2% NP40, 10% glycerol, protease inhibitor cocktail (Sigma), 1 mM PMSF] for 1 h at 4°C. The beads were washed 5× (10 mM Tris pH 7.5, 150 mM sodium chloride, 0.5 mM EDTA, protease inhibitor cocktail, 1 mM PMSF), eluted, boiled, separated and blots were probed with relevant antibodies.

For co-IP of over expressed protein HEK293T cells were transfected with GFP–INF2–WT,–E184K,–R218Q or–S186P and co-transfected with HACdc42QL (a gift from Harry Mellor, University of Bristol) or a transfection control plasmid. After 48 h cells were lysed in buffer [10 mM Tris pH 7.5, 150 mM sodium chloride, 0.5 mM EDTA, 2% NP40, 10% glycerol, protease inhibitor cocktail (Sigma), 1 mM PMSF], pelleted and the supernatant incubated with GFP-Trap agarose beads (Chromotek). After rotating at 4°C for 1 h the beads were washed 5× (10 mM Tris pH 7.5, 150 mM sodium chloride, 0.5 mM EDTA, protease inhibitor cocktail, 1 mM PMSF), protein was eluted in 50 μl sample buffer, separated, transferred and blots were probed with relevant antibodies.

For co-IP of G-actin, HEK293T cells were seeded into a six-well plate and transfected with FLAG–INF2–WT,–E184K,–R218Q,–S186P or transfection control. After expression, cells were lysed in 100 μl lysis buffer (50 mM Tris, pH 7.5, 120 mM sodium chloride, 1% NP40, 40 mM β-glycerophosphate, 1 mM benzamidine) and incubated on ice for 60 min to allow complete F-actin depolarization. INF2 was immunoprecipitated with anti-FLAG M2 (Sigma) and protein G magnetic beads (pre-blocked with 1% BSA) for 6 h at 4C. IPs were washed three times with 500 ml wash buffer (50 mM Tris, pH7.5, 120 mM sodium chloride, 0.5% NP40, 10% glycerol) and protein eluted by boiling with SDS sample buffer. Protein was resolved by SDS/PAGE and probed by western blot for INF2 (via FLAG-tag) and actin.

### Proteomics

GFP–INF2–WT was subcloned into pWPXL [pWPXL was a gift from Didier Trono (École polytechnique fédérale de Lausanne, Addgene plasmid # 12257)] and together with packaging vectors pMDG.2 and psAX2 [pMD2.G was a gift from Didier Trono (Addgene plasmid # 12259) and psPAX2 was a gift from Didier Trono (Addgene plasmid # 12260)] transfected into Lenti-X 293T Cell Line (Clontech). Human podocytes were transduced with GFP or GFP–INF2 lentivirus with 8 μg/ml polybrene overnight, the cells were thermo switched and differentiated for a minimum of 10 days [18]. 2× 175 tissue culture flasks were lysed and GFP and GFP–INF2 protein and interacting proteins were immunoprecipitated using the GFP-Trap system (Chromotek). Samples were separated on Nupage 4–12% precast gels (Invitrogen) and subjected to LC–MS/MS analysis on an Orbitrap Velos (Thermo) mass spectrometer as described previously [19,20]

### Immunofluorescence

Conditionally immortalized human podocytes stably expressing GFP–INF2 were seeded on to coverslips in a six-well plate or on to imaging dishes (MatTek) and differentiated for a minimum of 10 days. Cells were fixed in 4% paraformaldehyde (PFA) and permeabilized with 0.3% Triton TX-100 in PBS for 5 min. After blocking in 3% BSA cells were incubated in the relevant primary and secondary antibodies and mounted on slides in vectashield with DAPI (Vector Labs). Images were captured using a Leica AM total internal reflection fluorescence (TIRF) microscopy MC (multi-colour) system attached to a Leica DMI 6000 inverted epifluorescence microscope equipped with 405, 488, 561, 635 nm laser lines.

### Antibodies and other reagents

Mouse monoclonals: anti-HA (Covance), GFP (Roche), FLAG (Sigma); Rabbit polyclonals: anti-F-actin capping protein (Millipore), profilin 2 (Sigma), tubulin (Sigma), INF2 (Bethyl Labs, Millipore). Phalloidin-647 (Molecular Probes).

## RESULTS

**Figure 1:**
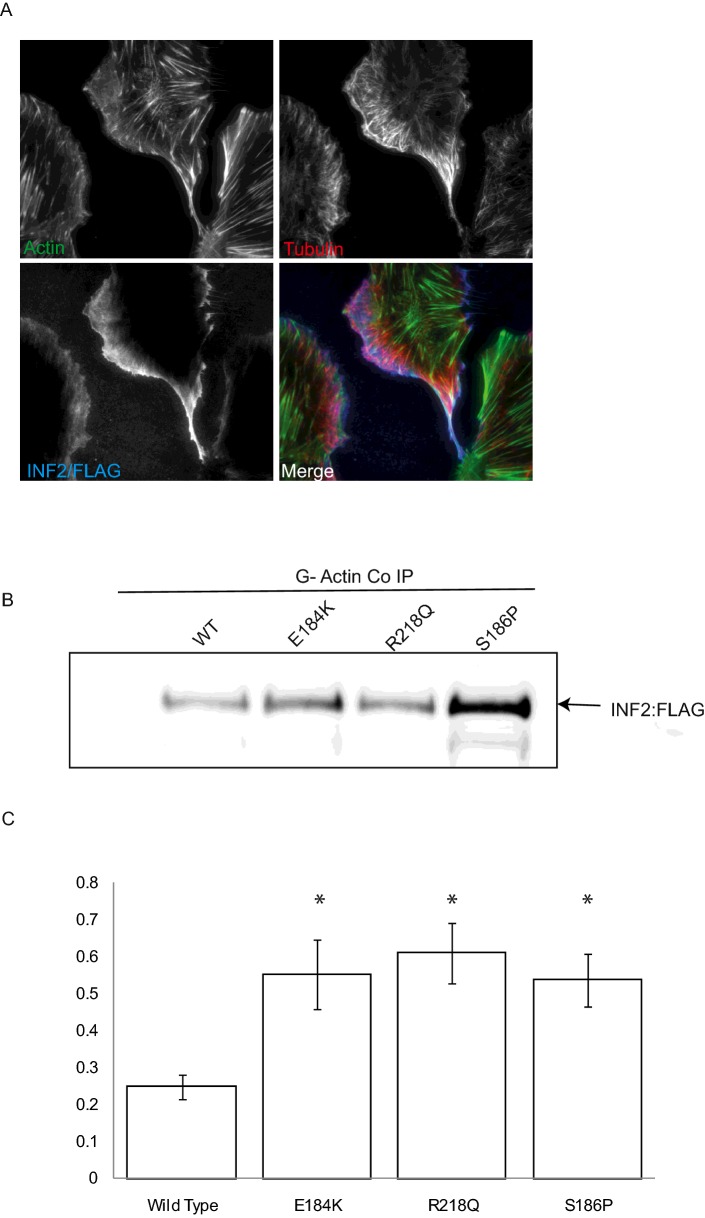
Localization of wild type INF2 and the interaction between INF2 and disease associated mutations with monomeric G actin (**A**) Cellular localization of INF2 in differentiated conditionally immortalized human podocytes. (**B**) Representative western blot of a co-IP between G-actin and FLAG-tagged wild-type and mutant INF2. (**C**) Graph of relative interactions between G-actin and the wild-type and mutant INF2. *n*=6, significance *P≤0.05.

**Figure 2:**
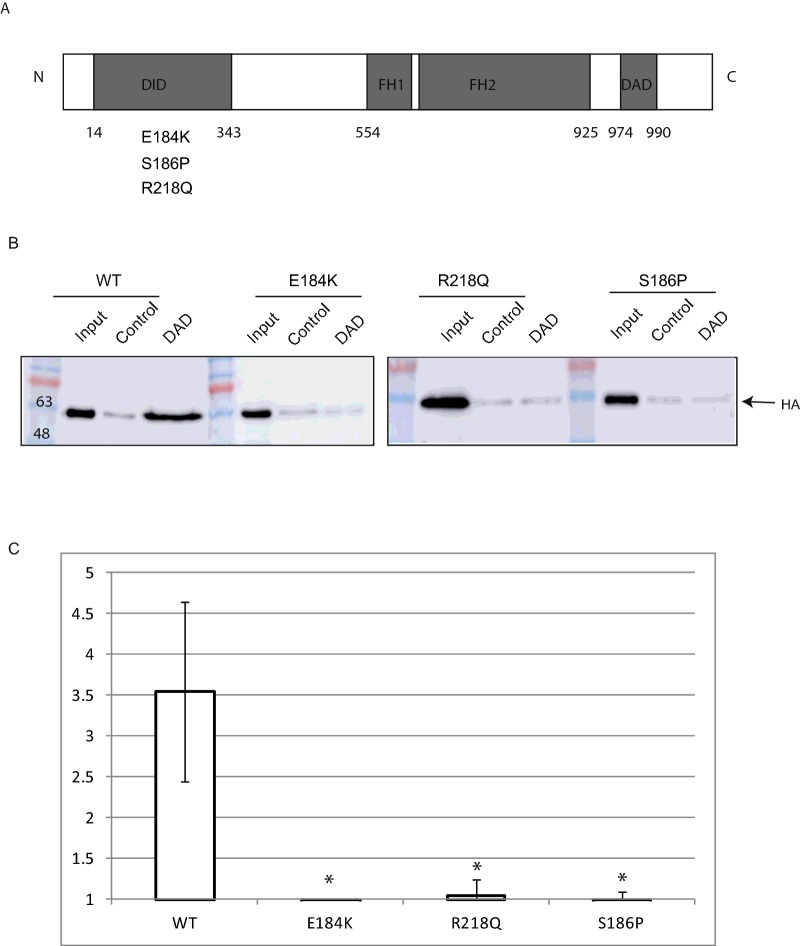
Interaction between wild type INF2:DAD wild type and disease associated mutations of INF2:DID (**A**) Schematic representation of INF2 showing the DID and the position of the E184K, R218Q and S186P mutants, the FH1 and FH2 domains and the DAD. (**B**) Representative western blot of a pulldown experiment between HIS:DAD immobilized on Co^2+^ agarose beads and wild-type and mutant HA-linked DID domains. (**C**) Graph of relative interactions between the INF2 DAD and the wild-type and mutant DID. *n* ≥ 4, significance *P≤0.05.

INF2 has been reported to localize to the endoplasmic reticulum (ER) [9]. In conditionally immortalized human podocytes we used TIRF to study the localization of INF2 near the plasma membrane and to exclude the ER pool. This demonstrated that there is INF2 at the cell periphery which co-localizes with both tubulin and actin (Figure 1A). Using Flag-tagged wild-type and three disease causing mutations in INF2 (the relative expression levels of the INF2 mutants is shown in Supplementary Figure S1) (E184K, S186P and R218Q) that all lie within the DID (Figure 2A) we show that INF2 interacts with actin and this interaction is increased in the presence of the disease causing mutations (Figures 1B and 1C). Actin has been shown to bind to the WH2 domain of INF2 which also acts as a DAD binding to the DID to auto-regulate INF2 activity [9]. Therefore to determine if this increase in actin binding was due to a loss of DID:DAD interaction we expressed and purified His-tagged INF2–DAD, linked the fusion protein to Co^2+^ beads and used these to pulldown HA-tagged INF2–DID–WT,–E184K,–R218Q and–S186P to measure the relative binding capacity of the wild-type and mutant DID domains with the DAD domain. Only the wild-type DID interacted with DAD. This demonstrated that in the disease causing mutations the DID:DAD interaction is disrupted (Figures 2B and 2C) and therefore the FH2 domain becomes accessible thus leading to the increased association with monomeric actin.

**Figure 3:**
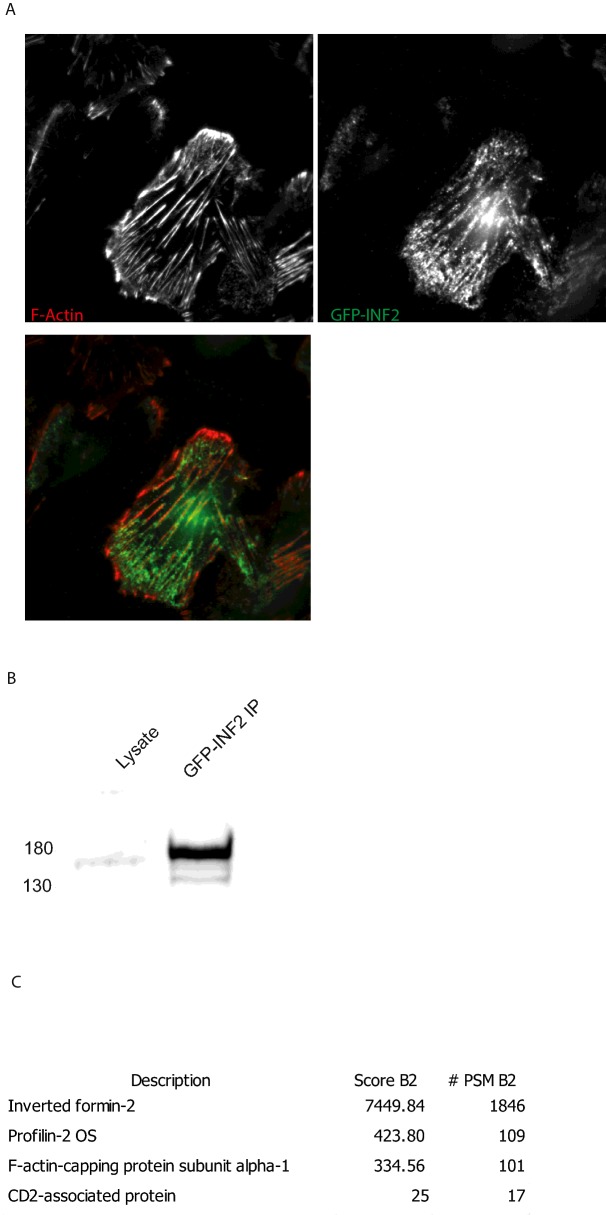
Expression of GFP-INF2 in Human podocytes and proteomic analysis of co-immunoprecipitated proteins (**A**) TIRF images of co-localization between GFP–INF2 and F-actin in differentiated ciPodocytes. (**B**) Western blot of an IP using GFP-Trap in differentiated ciPodocytes and probed with an anti-GFP antibody. (**C**) Selected proteins identified by MS that interact with GFP–INF2 in differentiated ciPodocytes.

**Figure 4:**
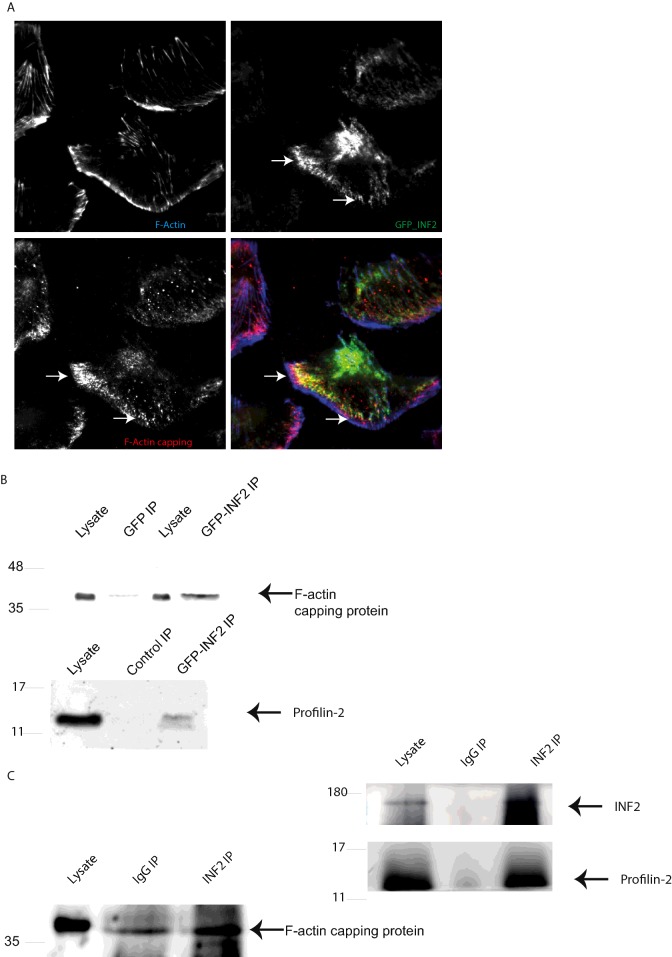
TIRF imaging of GFP-INF2 in differentiated podocytes, co-immunopreciptiation of GFP tagged and endogenous proteins with GFP-INF2 or endogenous INF2 (**A**) TIRF images of co-localization between GFP–INF2WT and F-actin capping protein in ciPodocytes. (**B**) Western blots of IPs using GFP-Trap in differentiated podocytes probed with an F-actin capping protein antibody and a profilin 2 antibody. (**C**) Western blot of a co-IP of endogenous protein using an INF2 antibody linked to agarose beads and probed with antibodies to INF2, F-actin capping protein and profilin 2.

**Figure 5:**
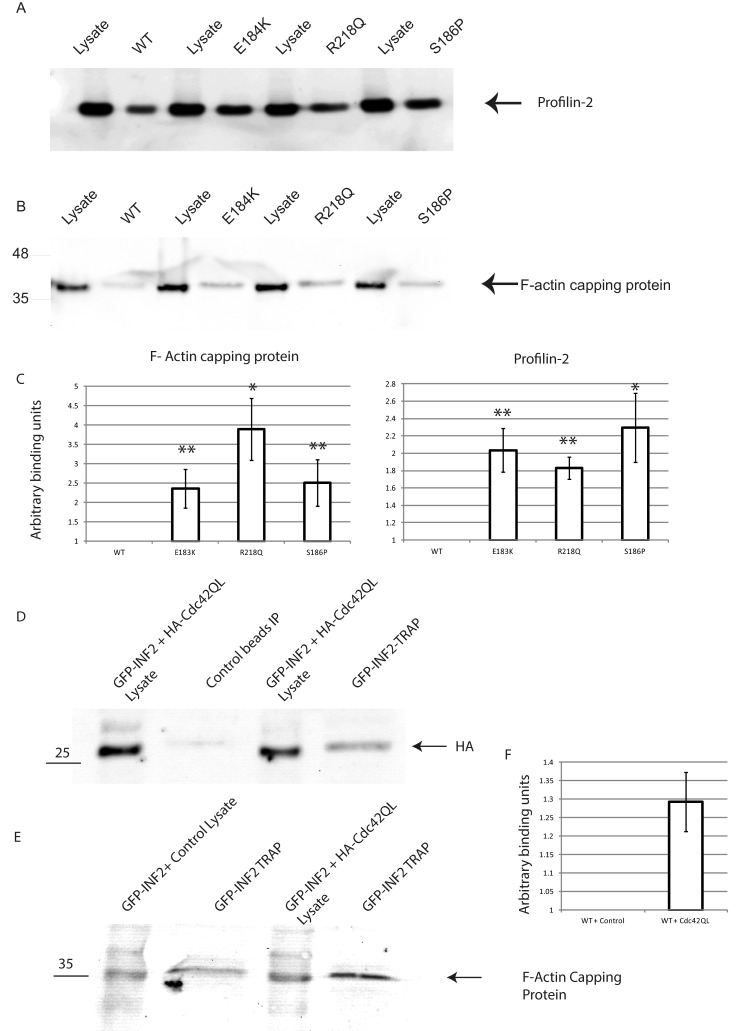
Quantification of the interaction between profillin 2 and the F-actin capping protein with wild type and disease causing mutations of INF2 (**A**) Representative western blots of the co-IP of profilin 2 and either GFP–INF2WT, GFP–INF2E184K, GFP–INF2R218Q or GFP–INF2S186P. (**B**) Representative western blots of the co-IP of F-actin capping protein and either GFP–INF2WT, GFP–INF2E184K, GFP–INF2R218Q or GFP–INF2S186P. (**C**) Graphical representation of the relative amount F-actin capping protein and profilin 2 that co-IPs with GFP–INF2WT, GFP–INF2E184K, GFP–INF2R218Q or GFP–INF2S186P normalized for expression levels and relative to GFP–INF2WT which is set at 1. *n* ≤ 3. (**D**) Western blot of a co-IP of GFP–INF2WT and HACdc42QL. (**E**) Representative western blot of the co-IP of F-actin capping protein co-transfected with control or HACdc42QL. (**F**) Graph of the relative amount of F-actin capping protein that co-IPs with GFP–INF2WT + control and GFP–INF2WT + HACdc42QL normalized to GFP–INF2WT + control which is set at 1. *n* ≥ 4, significance *P≤0.05, **P≤0.01.

In order to further explore the effect of these mutations on INF2 biology we expressed GFP-tagged INF2 in podocytes using lentiviral transduction. GFP–INF2 co-localizes with F-actin as expected in differentiated podocytes so we were confident that the fusion protein is properly localized (Figure 3A). These and control cells, expressing GFP only, were then lysed and the GFP immunoprecipitated using the highly efficient GFP-Trap method [21]. The precipitated GFP and GFP–INF2 were separated by SDS/PAGE and interacting proteins analysed by LC–MS/MS after in-gel tryptic digestion. The MS analysis identified two proteins, profilin 2 and F-actin capping protein (CapZ α-1), that were significantly more abundant in the GFP–INF2 pulldown compared with the GFP control (Figures 3B and 3C). To confirm the MS results further pull downs were undertaken using both overexpressed and endogenous INF2 in podocytes showing that indeed INF2 interacts with both proteins (Figures 4B and 4C). Furthermore TIRF microscopy in human podocytes showed co-localization of INF2 with F-actin capping protein at the periphery of the cell (Figure 4A). We used F-actin capping protein and profilin pull down as a readout for INF2 activity as it has been previously reported that profilin binds to the FH1 domain of mDia [22] and we speculated that the loss of interaction between the DAD and mutant form of DID will lead to activated INF2 and therefore increased interaction with target proteins. This proved to be the case as the mutant forms of INF2 pulled down significantly more F-actin capping protein and profilin than the wild type (Figures 5A–5C).

INF2 is reported to bind to the Rho-GTPase CDC42 and the mutant forms of INF2 are reported to show increased binding to this protein [11,16,17]. We co-expressed GFP–INF2 with the active form of cdc42 (cdc42QL [23]) and demonstrated that there was an interaction (Figure 5D). Furthermore we showed that the presence of the active cdc42 resulted in a significant increase in the interaction between wild-type INF2 and the F-actin capping protein (Figure 5E).

## DISCUSSION

Focal segmental glomerulosclerosis (FSGS) is a devastating form of nephrotic syndrome. The aetiology of FSGS is still unknown although inherited forms of the disease are now providing revolutionary clues to the underlying pathogenesis and target the glomerular podocyte [4,24]. Podocytes are the final layer in the kidney's glomerular capillary wall. Together with the basement membrane and glomerular endothelial cells they form the barrier through which filtration occurs. Podocytes play an essential role in preventing proteinuria and are an important target in the pathogenesis of renal disease. Podocytes have a remarkably elaborate and highly specialized morphology that is dependent on the actin cytoskeleton and which is essential for maintaining glomerular function and integrity in healthy kidneys [1]. There is compelling evidence that podocytes display a limited physiological motility, and that changes in podocyte motility may underlie nephrotic disease [1,25]. Thus it is clear that the specialized function of the podocyte in the normal kidney depends critically on an underlying network of dynamic and interconnected actin and microtubule polymers. The mechanism through which this morphology is achieved and maintained in the normal kidney is not currently understood.

Mutations in the DID domain of the formin family member, INF2, have recently been associated with FSGS [3–7] and in Charcot–Marie–Tooth disease with glomerulopathy [16]. Formins are a highly conserved family of large multi-domain proteins that play essential roles in the regulation of actin and microtubule cytoskeletons [26]. Interestingly certain of these mutations only result in a renal phenotype suggesting that these cause a disruption of podocyte morphology as a result of aberrant regulation of actin dynamics [16]. Little is known about the role of the INF2 DID domain, although one study has shown that association of INF2 DID domain with the DAD domain of the related family member, mDia1 inhibited RhoA-mDia-dependent actin polymerization and serum response factor regulated gene transcription [14].

We have shown that recombinant, truncated INF2 regions comprising the DID and DAD domains interact *in vitro* and that FSGS associated mutations (E184K, S186P and R218Q) reduce the affinity of this interaction. The mutant forms of INF2 also show an increased association with monomeric actin as shown by co-IP. This would be consistent with the auto-regulation of other formins whereby interaction of the DID and DAD domains constrains INF2 in a closed conformation inhibiting its actin nucleating and polymerizing activities. Loss of this auto-inhibition through loss of affinity of the DID and DAD interaction leads to revealing of the WH2 domain and increased actin binding. This is in agreement with previous data showing that disruption of the DID/DAD interaction causes constitutive actin polymerization by INF2 in cells [17].

Using a combination of GFP–INF2 expression in human podocytes and GFP-Trap purification coupled with MS we identified profilin 2 and the F-actin capping protein, CapZ α-1 as interactors of INF2. These interactions were confirmed using both expressed and endogenous INF2. Importantly these interactions are increased by the presence of the disease causing mutations and by the co-expression of an active CDC42 construct. CDC42 is a known regulator of INF2 so this data suggest that both the mutations and cdc42 lead to a decrease in the DID/DAD interaction increasing the binding of actin, profilin 2 and the F-actin capping protein [11].

Profilin is a known interactor of the formin family and has been shown to regulate the effects of these proteins on actin dynamics [27–29]. Indeed profilin has recently been shown in fission yeast cells to regulate the F-actin network by favouring formin over the Arp2/3 complex [30]. INF2 and profilin have been shown to regulate the assembly and turnover of short actin filaments [31]. Therefore the increased binding of profilin to INF2 in the presence of the disease causing mutations is likely to have a significant effect on the regulation of podocyte actin dynamics. Furthermore we have demonstrated that INF2 also binds to CapZ α-1 and that like profilin this binding is also increased in the presence of the disease causing mutations. Actin capping proteins are key regulators of actin dynamics and formins have been shown to antagonize the actions of these proteins [32,33]. Interestingly, in fission yeast during cytokinesis profilin has been shown to mediate the competition between capping protein and formin [34]. Therefore this again suggests that the FSGS causing mutations will disrupt the tight regulation of the podocyte actin cytoskeleton.

Apart from its role in the regulation of actin dynamics INF2, like other formins, has been shown to bind and have effects on microtubules [35] and is thought that formins may act to co-ordinate actin filaments and microtubules which is essential for many cellular processes [26,36]. In cultured podocytes INF2 regulates cellular actin dynamics by antagonizing Rho/diaphanous-related formin signalling and disease causing mutations have been shown to alter this signalling in the glomerulus [14,15]. mDia mediates Rho-regulated formation and orientation of stable microtubules and actin-capping protein promotes microtubule stability by antagonizing the actin activity of mDia [37,38]. Therefore this suggests that actin capping protein may function by co-ordinating cross-talk between actin and microtubules and this may be disrupted by the disease causing INF2 mutations. In support of this INF2 has been shown to organizes lumen and cell outgrowth during tubulogenesis by regulating both F-actin and microtubule cytoskeletons and the authors of the present paper suggested that the effects of disease causing INF2 mutations may be via alternative or additional mechanisms to altered actin regulation namely via effects on microtubular dynamics [39].

CapZ has been shown to interact with CD2AP which plays a key role in the maintenance of the podocyte slit diaphragm [40] and INF2 has been shown to bind to nephrin and to regulate lipid raft-mediated lamellipodial trafficking of slit diaphragm proteins [14]. Interestingly CD2AP was identified as a binding partner of INF2 in our proteomic screen although this is still to be confirmed (Figure 3). Nephrin and the slit diaphragm complex are known to be a crucial regulators of the podocyte cytoskeleton and therefore our data suggest that INF2 binding to profilin and CapZ α-1 may play a critical role in the tight regulation of podocyte actin and microtubular dynamics via interaction with the podocyte slit diaphragm and this is altered in the presence of disease causing mutations [1].

## Abbreviations

co-IP: co-immunoprecipitation
DAD: diaphanous auto-regulatory domain
DID: diaphanous inhibitory domain
ER: endoplasmic reticulum
FH1 and 2 domain: formin homology 1 and 2
FSGS: focal segmental glomerulosclerosis
INF2: inverted formin 2
TIRF: total internal reflection fluorescence
WH2 domain: Wiskott–Aldrich syndrome homology region 2
